# Physical and functional aspects of persons with multiple sclerosis practicing Tai-Geiko: randomized trial

**DOI:** 10.6061/clinics/2020/e1272

**Published:** 2020-01-06

**Authors:** Viviane Regina Leite Moreno Ultramari, Adriano Percival Calderaro Calvo, Rosilene Andrade Silva Rodrigues, Waléria Christiane Rezende Fett, Jose Urias de Moraes Neto, Almir de França Ferraz, Michelle Jalousie Kommers, Heloise Helena Siqueira Borges, Michell Vetoraci Viana, Monica Cattafesta, Luciane Bresciani Salaroli, Carlos Alexandre Fett

**Affiliations:** I Nucleo de Estudo em Aptidao Fisica, Informatica, Metabolismo, Esporte e Saude (NAFIMES), Universidade Federal do Mato Grosso, MT, BR; II Programa de Pos Graduacao em Desempenho Humano Operacional (PPGDHO), Universidade da Forca Aerea (UNIFA), Rio de Janeiro, RJ, BR; III Grupo de Estudos em Actividade Fisica e Promocao da Saude, Departamento de Graduacao em Educacao Fisica, Universidade Sao Judas Tadeu (USJT), Sao Paulo, SP, BR; IV Programa de Pos Graduacao em Saude Coletiva (PPGSC), Centro de Ciencias da Saude, Universidade Federal do Espirito Santo (UFES), Vitoria, ES, BR

**Keywords:** Muscular Strength, Postural Balance, Tai-Geiko

## Abstract

**OBJECTIVES:**

This study aimed to verify the influence of Tai-Geiko on the physical and functional aspects of people with multiple sclerosis (MS).

**METHODS:**

This was a parallel-group, randomized trial with two arms. People with MS were allocated to an experimental group (EG) (n=10) and control group (CG) (n=09). The participants received multidisciplinary care supervised by a physiotherapist in the Tai-Geiko exercise. Participants underwent the assessments after the intervention. The Expanded Disability Status Scale (EDSS-maximum score of 6.0), strength test (kgf) using a dynamometer, Timed Up and Go mobility test (TUG), and stabilometric balance test (Platform EMG system^®^) were evaluated. Demographic data were recorded, including age, sex, comorbidities, lifestyle and classification of MS. Clinical Trials (ReBeC): RBR-4sty47.

**RESULTS:**

The EG group improved in 12 variables, and the CG improved in 3 variables. The following values were obtained for pre/postintervention, respectively: EG: lumbar force (38/52 kgf), TUG (11/9 s), locomotion velocity (519/393 ms); double task two (53/39 s); platform stabilometric trajectory: traversed get up (39/26 s) and sit (45/29 s); anteroposterior (AP) amplitude rise (11/8 cm) and sit (12.40/9.94 cm) and anteroposterior frequency rise (1.00/1.56 Hz) and sit (0.8/1.25 Hz) (*p*<0.05); CG: right-hand grip force (26/29 kgf); TUG (9.8 /8.7 s) and AP (11.84 /9.53 cm) stabilometric amplitude at the sitting moment (*p*<0.05), (3.2/5.99 Hz, p=0.01) and sit (3.47/5.01 Hz, *p*=0.04).

**CONCLUSION:**

Tai-Geiko practice can be suggested as complementary exercise in the rehabilitation of persons with MS.

## INTRODUCTION

Multiple sclerosis (MS) is a chronic and progressive disease that affects the central nervous system (CNS) and is characterized as a demyelinating and inflammatory axon injury (1-3); it is more common in women, especially in middle-aged women, than in men (4,5). The World Health Organization (WHO) (6) considers MS to be a public health problem since it has a high-cost treatment, high disability rate, and premature mortality. It is estimated that there are 2.5 million people with MS in the world, and in Brazil, it is considered a rare disease with a low prevalence, affecting approximately 13,000 Brazilians with MS disease under treatment (7,8).

The etiology of MS is still unknown; the genetic predisposition associated with environmental exposures increases the number of T lymphocytes in the bloodstream, which deregulates the immune process (9). In the initial phase of MS, the myelin sheath deteriorates, which triggers a progressive interruption of the electrical impulses (3) and leads to a neurological incapacity. Thus, movements are altered due to the decrease in muscle strength and balance, making the basic movements in daily living difficult and compromising the quality of life (10). Thus, it is of the utmost importance that people with MS be oriented and encouraged to maintain a healthy lifestyle to avoid falls and exclusion from social life and from activities that require physical effort (11-13).

Tai-Geiko is an Eastern Bioenergetic Gymnastics program that seeks to work the human organism in a holistic way, namely, from body to mind, through soft exercises. Among the types of physical exercise aimed at the rehabilitation of individuals in a global way, this practice can be carried out according to the individual’s physical ability if it is performed mildly. Moreover, it can delay the aggravation of MS since it is founded on the basic activities of a person’s daily life. Therefore, Tai-Geiko is proposed to maintain the muscle strength and improve the balance and cognition of healthy or sedentary people (14). Tai-Geiko also improves emotional control because it focuses on affectivity in order not to limit the independence or functional autonomy of the individual (14). However, there are no scientific studies on Tai-Geiko that confirm the effectiveness of this technique in people with MS.

Therefore, this study aimed to verify the influence of Tai-Geiko on the physical and functional aspects of individuals with MS. The research questions for this randomized controlled trial were as follows: (1) Does physical exercise with the Tai-Geiko technique improve the physical-functional aspects of people with MS? (2) Do people practicing the Tai-Geiko technique improve the type of body balance that is measured by the force platform?

## MATERIALS AND METHODS

### Design

**Figure 1:**
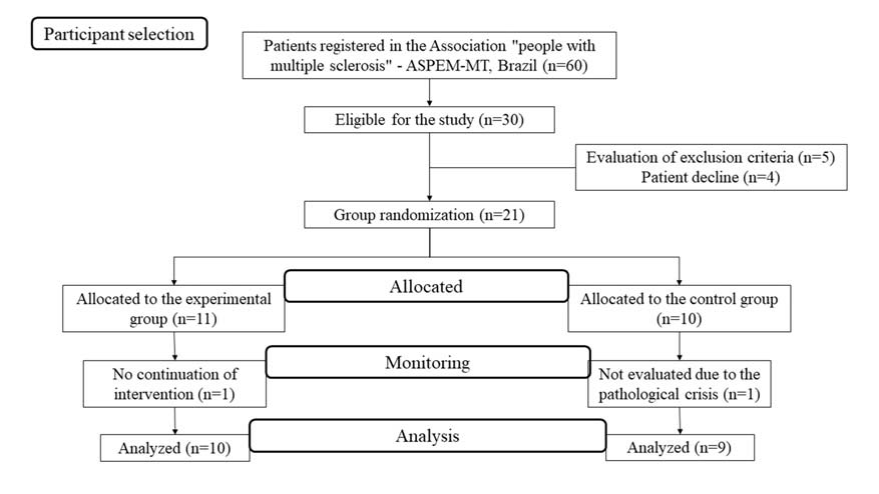
Recruitment, attribution, monitoring and analysis of participants. EDSS = Expanded disability status scale.

This experimental study was a parallel, randomized controlled trial with a Tai-Geiko physical-exercise intervention. Participants were selected from the population of people with MS in Mato Grosso, Brazil (ASPEM-MT), which was composed of 60 registered patients of both genders. Among them, only 30 individuals were eligible for this study, according to the Expanded Disability Status Scale (EDSS ≤6 (Figure 1).

### Participants, therapists, centers

All participants had a diagnosis of MS confirmed by a neurology physician at least 12 months before the experiment, and their inclusion in the research was performed according to the EDSS (15), which was used to assess the mobility, disease symptoms, emotional state, personal satisfaction, thinking and fatigue, and family situation of the individual. Data were obtained in two stages by a questionnaire and a physical evaluation. In the form, the participants answered questions on sociodemographic aspects. The time locomotion speed was determined according to the EDSS.

For inclusion, all participants had to have a score less than 6.0 (EDSS) (“walking independently without the aid of a walking stick or crutch”) and be under medical supervision. The exclusion criteria were as follows: no medical diagnosis of diseases secondary to MS, such as physical or mental disabilities, heart diseases, dyslipidemias, and diabetes mellitus.

The Tai-Geiko exercise technique was performed by a physical education professional and physiotherapist throughout the intervention period of the study.

### Intervention

The participants were randomly divided into two groups (control and experimental). However, four of them refused to participate in the study, 5 were excluded by the research criteria explained below, 1 gave up before the conclusion of the exercise protocol, and 1 could not be reevaluated due to an episodic outbreak of MS. After randomization, the experimental group (EG) waited one month to start Tai-Geiko practice, and the CG maintained its usual routine. Therefore, the final sample consisted of 9 patients in the control group (CG) and 10 patients in the EG practicing Tai-Geiko twice a week for one hour for a period of eight weeks. The intervention protocol can be found in Table 1.

### Outcome measures

A complete anamnesis on the practice of habitual physical bodybuilding, sociodemographic antecedents and state of general health was performed.

The evaluations were performed by the research team, and training in Tai-Geiko was conducted by the physical education professional. Participants were assessed by the EDSS, which quantifies the disabilities occurring in functional systems during the course of MS, the score from 0 (normal neurological examination) to 7.5 (restricted to wheelchairs or beds) (15). The speed of locomotion in time is associated with the EDSS scale, which was timed, and how long in seconds the patient takes to walk independently on a trajectory (15).

Prior to the main test, participants performed the test to familiarize themselves with the protocol.

The strength test (kgf) using a dynamometer (16) was repeated three times, and the average of the measurements was calculated. Right and left hand scapular, lumbar and manual strength were evaluated (16,17).

The Timed Up and Go mobility test (TUG) assesses mobility, locomotor performance, and balance change. It consisted of timing the movement in a 3 meter course, moving from sitting to standing and walking, in which taking less than 20 seconds to perform corresponded to a low risk of falling; 20 to 29 seconds, a medium risk of falling; and 30 seconds or more; a high risk of falling (18).

The dual cognitive-motor task was aimed at verifying possible alterations in cognition and/or motor skills; the average time needed for each participant to perform the assigned task of wearing a shirt, buttoning buttons and pronouncing female names was calculated (19,20).

The Platform EMG system^®^ stabilometric equilibrium test covered the Center of Pressure (CoP) trajectory, CoP AP amplitude, CoP medial-lateral (ML) amplitude and elliptical area; the lower the score, the better was the participant’s balance. However, the CoP AP median frequency and CoP ML median frequency were higher when the score was greater than the equilibrium area) (21).

Demographic data were recorded, including age, sex, comorbidities, lifestyle and classification of MS.

Balance was measured using the force platform, which aims to evaluate the static and dynamic posture and is an instrument considered objective and sensitive to balance deficits. The test was performed with a chair with an adjustable height, which was positioned on the platform. The participants were requested to remain in the orthostatic position on the platform and perform the movement of getting up and sitting down. Moreover, they were instructed to continue with their usual activities and report any symptoms or presence of crisis due to pathology.

The primary endpoint was that at which the physical exercise Tai-Geiko presented/demonstrated improvement of balance. The lateral medial balance showed that a measurement above 3 cm indicated a risk of falling, and an anteroposterior (AP) measurement above 10 cm increased the risk of falling. These data were derived from the force platform.

### Ethical issues

The Research Ethics Committee of the Health Sciences Center of the Hospital Universitário Julio Müller (HUJM-UFMT) approved the study under opinion N° U1111-1196-2811 (CAAE: 52661316.5.0000.5541). The study was registered in the Brazilian Registry of Clinical Trials (ReBeC) under number RBR-4sty47. All participants in the study signed an informed consent form, and the investigation was conducted in accordance with the Declaration of Helsinki.

### Data analysis

The sample was adopted by the formula below:


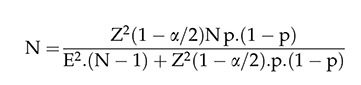



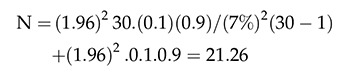


where, Z ^2^ (1-α/2) corresponds to the value of the 95% confidence interval and the error used was 7%, which corresponds to the difference between the proportion of the true MS population and the estimated proportion of individuals with MS; P = the proportion of patients with this pathology; N = total number of individuals with sclerosis that could participate in the research. Therefore, the 19 people who were evaluated corresponded to 63.3% of the total patients in the sample.

Data normality was determined by the Shapiro-Wilk test for the pre- and postintervention intergroup analysis. The sample calculation used the total census base of all patients diagnosed with MS in the state; there was a loss of 20% of the total participants in the study, namely, 10% in the EG and 10% in the CG. The T-test was used for parametric distributions, and the Mann-Whitney test was used for nonparametric distributions, whereas for the intragroup analysis, the paired t-test was used for parametric distributions, and the Wilcoxon test was used for nonparametric distributions. A value of *p*<0.05 with the 95% confidence interval was considered significant.

## RESULTS

In total, 19 people with MS participated in this study (5 men and 14 women, aged between 14 and 59 years [44.89±10.71 years]). The frequencies of the types of sclerosis that comprised the sample were 84.2% remitting recurrent MS, 10.5% progressive primary MS, and 5.3% progressive secondary MS. After the intervention, for data analysis, 9 patients remained allocated to the CG (disease period of 5±2.94 years), and 10 patients remained allocated to the EG (disease period of 9.1±6.09 years). The participants in the EG were not completely sedentary, 1 had a regular walking practice and underwent physical therapy, and another 1 subject participated in street races. Individuals in the CG practiced other types of exercises: 2 of them performed Pilates, 2 exercised in the gym, and 2 underwent traditional physiotherapy.

There was a significant difference with respect to the right-hand grip strength in the CG and the lumbar strength in the EG. There was no significant difference between the groups (Table 2).

Regarding the evaluations of physical and cognitive performance, there were no significant differences; only the dual cognitive-motor task (DT2) variable showed differences between the groups at the preintervention time, but it was equalized after the intervention. However, in the comparison of the pre- and postintervention time, the EG showed a significant difference after the intervention in the following variables: TUG, locomotion speed, dual motor task one (DT1), and dual motor task two (DT2) (Tables 3 and 6).

According to the data below, only the variable CoP ML amplitude showed a significant difference after the intervention. However, in the comparison of the pre- and postintervention time, the EG presented a significant difference in the CoP trajectory covered, CoP AP amplitude (AP), CoP AP median frequency, and CoP ML median frequency (Table 4).

Regarding the stabilometric variables of sitting motion**,** there was a significant difference only for the CoP ML amplitude variable after the intervention. However, in the comparison of the pre- and postintervention, the EG presented significant differences for the CoP trajectory covered, CoP AP amplitude, and CoP AP median frequency (Table 5).

### Adverse effects on health

One trial participant entered the outbreak ward because of personal emotional issues, and the neurologist decided to remove him from the trial.

The EG participation in the practice of Tai-Geiko for MS consisted of presence above 75% and tolerance monitored by the Borg scale (value up to 8). Membership consisted of providing an extra day to replace absences in the exercise program.

## DISCUSSION

The EG exhibited an improvement in strength, displacement speed, cognitive ability, and balance. The EG had 12 variables improved, whereas the CG showed improvement in only 3 of the 22 evaluated physical performance variables. Moreover, regarding balance, the loss of which is a significant disability attributed to MS, the EG showed results significantly better than those observed in the CG in the final evaluation for the measurement of the ML amplitude, which is specific and determinant for the improvement of this physical variable. There was a significant difference between the groups in the primary endpoint (i.e., physical activity of mild to moderate intensity improved in the EG after 8 weeks at the midlateral balance, with a *p* value of 0.04).

Regarding scapular and hand muscle strength, there was no significant difference between the groups, and no change was verified in the EG. These results can be partly explained by the fact that Tai-Geiko is an exercise of mild to moderate intensity and is not specifically aimed at the strength of upper limbs (14). The improvement in the CG only in hand grip may have occurred due to the practice of other physical activities, such as Pilates, weight training, and physical therapy for MS. These activities were performed twice a week, totaling 120 min weekly, on average. EDSS is a measure that reports the progression and consequent worsening of MS symptoms, and in this case, the EG started participating in this study with a score that was half a point higher (classified as having minimum disability), and the CG was classified as without disability. Therefore, it was expected that the trainability of the strength variable was more compromised in the EG (16). In this sense, the increase in strength even without significance is an important result for the EG because a decrease in this variable with disease progression was expected.

During the Tai-Geiko protocol, the EG did not undergo physiotherapeutic treatment or other physical activity.

There was an increase in lumbar muscle strength only in the EG. This may be justified by the effect of learning motor and cognitive control techniques specific to Tai-Geiko (22,23). This practice requires many postures of isometric maintenance and respiratory control, consciously activating the muscles in the middle of the body and pelvic girdle muscles. Thus, this modality potentiates the neuromuscular stimuli with alterations of intensity associated with conscious control of postures, offering stimuli that can compensate for the slowing of nerve impulses (24).

The present study lasted eight weeks of intervention, supported by findings that showed that eight- to twelve-week progressive loading training can increase muscle strength in people with MS ([Bibr B25] 26-28). On the other hand, it has been demonstrated that high-intensity training can (27) improve strength but is not effective in the balance of people with MS (29). Thus, Tai-Geiko, as a proposal for light to moderate global activity, has shown responsive stimulation sufficient for the performance of both strength and balance in people with MS. Quite possibly, characteristics of body postures with different levels of coordination and balance required by the Tai-Geiko techniques positively impacted these variables.

In the EG, after the intervention, the Tai-Geiko exercise improved the aspects of functional and cognitive ability. In these patients, the improvement may be correlated with the balance since it makes the patient more confident in walking. Similarly, other authors (30-33) reported an improvement in the walking speed and balance after aerobic exercise, positively interfering with agility and dual task performance. Thus, in our study, it was possible to observe an improvement in the dual task in the intragroup analysis after the Tai-Geiko intervention.

The EG had an improvement in the TUG and in the two dual task tests, whereas the CG only had an improvement in the TUG. Although there was no difference in the intragroup comparison, these results demonstrate that Tai-Geiko had an important impact on cognitive and learning aspects in the EG compared with the CG. Other studies have shown that these improvements occur after 12 weeks of physical exercise in dual task tests (19). Thus, the result found in our study becomes more relevant, given that in only 8 weeks of Tai-Geiko practice, important changes occurred in these variables, which could be more prominent in a longer intervention time.

The standing and sitting test is considered simple, but it can be a useful tool to assess the consequences of impaired balance in people with MS (21). In our study, this test was performed on a force platform to make the standing and sitting analysis more sensitive with a stabilometric measure (21). Thus, regarding the stabilometric variable, the AP amplitude in the standing stage was improved in the EG, possibly because the Tai-Geiko stimulates the body consciousness interiorization when the standing motion is performed (14). Regarding the sitting stage, the AP amplitude was improved in the two groups. The sitting motion is more passive and does not require as much body consciousness to initiate the motion, and it is aided by the action of gravity. This fact can be explained by the motor learning from the test, which improved the retesting on the force platform.

Change in the ML amplitude is one of the main deficits in people with MS, leading to an increase in the postural balance due to balance disturbances that are adjusted by the hip joint after activating the abductor and adductor musculature (34). The results presented here are consistent with those in the previous literature since only ML amplitude showed improvement in the Tai-Geiko EG. This balance recovery works in conjunction with the sensory, visual, and proprioceptive systems (34,35), which may have been stimulated by the experimental protocol, considering the fact that Tai-Geiko is performed with proprioceptive and visual exercises combined with meditation. Thus, movements performed during Tai-Geiko practice contributed to strengthening the pelvic girdle, which stabilizes and harmonizes the ML displacement, resulting in better balance and consequently better confidence in walking and performing the activities of daily living.

The number of people with MS who could be included in the research was one of the limiting factors in this study. Additionally, the studied sample universe was accessed in its entirety, but we did not have a larger contingent that allowed us to use the simple draw criterion since there were no more individuals to be allocated to the research. Additionally, people from the CG practiced other activities, such as Pilates and regular physiotherapy, which could have interfered with the results; moreover, due to ethical reasons, the routines of this group were not changed. On the other hand, it is very difficult to obtain homogenous groups of people with MS because each individual has specific characteristics relating to this disease, and they participate in distinct programs of rehabilitation. This fact demonstrates the importance of protocols that can provide proven benefits for this population.

## CONCLUSION

The Tai-Geiko practice can be suggested as a complementary exercise in the rehabilitation of people with MS, aiming at improving functional ability and contributing to motor and cognitive performance. Thus, Tai-Geiko allows people with MS to not limit themselves from carrying out activities of daily and social living due to fear of falling and exclusion.

## AUTHOR CONTRIBUTIONS

Ultramari VRLM, Calvo APC, Rodrigues RAS, Fett WCR, Moraes Neto JU, Ferraz AF, Kommers M, Borges HHS, Viana MV, Cattafesta M, Salaroli LB and Fett CA contributed to the conceptualization, data curation, formal analysis, funding acquisition, investigation, methodology, project administration, resources, supervision, validation, visualization, writingoriginal draft, and writing-review and editing.

## Figures and Tables

**Table 1:** Characterization of base homogenization of people with multiple sclerosis.

Variables	Total sample (n=22)	Control group (n=11)	Experimental group (n=11)
Gender (n, %)			
Female	16 (72.7%)	08 (72.7%)	08 (72.7%)
Male	06 (27.3%)	03 (27.3%)	03 (27.3%)
Marital status (n, %)			
Single	09 (40.9%)	04 (36.4%)	05 (45.5%)
Married	12 (54.5%)	06 (54.5%)	06 (54.5%)
Divorced	01 (4.5%)	01 (9.1%)	-
Physical activity practice (n, %)			
Yes	11 (50%)	06 (54.5%)	05 (45.5%)
No	11 (50%)	05 (45.5%)	06 (54.5%)
Clinical classification (n, %)			
Recurrent sclerosis	18 (81.8%)	09 (81.8%)	09 (81.8%)
Progressive primary	02 (9.1%)	-	02 (18.2%)
Progressive secondary	02 (9.1%)	02 (18.2%)	-
EDSS (scale) (M±SD)	2.38 (1.10)	2.40 (1.13)	2.36 (1.12)
Age (years) (M±SD)	34.09±10.52	31.81±8.61	36.36±12.13
Disease period (years) (M±SD)	6.81±5.15	4.9±3.01	8.72±6.21
Height (meters) (M±SD)	1.62±0.07	1.61±0.07	1.64±0.08

M = mean; SD = standard deviation; EDSS = Expanded Disability Status Scale**.**

**Table 2:** Training protocol for intervention in people with multiple sclerosis.

Phases	Training protocol	Intensity
Heatment 10 min	Performed dynamically, combining walking in all the directions (front, side and back and elongation)	Scale of Borg, (intensity light).
Exercises Tai-Geiko 40 min	**1. Lying hip lift:** dorsal decubitus position on the mat (ground); legs flexed at 90 degrees; they extended the hip keeping the shoulders and arms on the ground, contracting the gluteal muscles and abdomen; the movement went up to the hip to align with the spine and then returned to the initial position. **2. Squat:** orthostatic position; feet slightly wider than shoulder width; they flexed their knees at approximately 90 degrees, returning to the initial position. **3. Balancing exercises:** standing balance will be worked on, in which the people will stay with only one foot fixed on the ground and the other suspended, first with open eyes and then with closed eyes. **4. Lumbar flexion:** orthostatic position; feet farther than the shoulders; the trunk flexed forward until the hands touch the ground and then a return to the initial position; with use of the stick. **5. Therapy Ryoho:** 15 min, introduction to the Shiatsu (Japanese method of finger pressure massage by the body). **6. Chi Kung:** ratios extended along and lateral to the body; flexed elbows in their full range; return to the starting position. **7. Exercise Kokyu** (the practice of controlled abdominal breathing, made by nasal inspiration and oral expiration) 10 min.	Scale of Borg), range between 3 and 5 (moderate to strong intensity) Scale perceived exertion).
Return the calm 10-15 min	The postexercise Tai-Geiko was realized by the stretching of the muscle groups worked, and the participant was guided in meditation. **Inspired exercises:** seated position for the observation of the movements of nature, animals and plants, where all senses (smell, taste and hearing) were exercised (Chi Kung).	Scale of Borg, (intensity light) Scale of perceived exertion.
Local	Airy room with air conditioning and a dimension of 10 x 15 meters with a mat on the floor, at the Faculty of Physical Education of Federal University.	-
Number of exercises	9 (nine) in total. Average time of approximately 140 min per week	-

**Table 3:** Anthropometric and physical performance characteristics of people with multiple sclerosis.

Variables		Experimental (EG)					Control (CG)					Intergroups	
												(EG) x (CG)	
		Preintervention		Postintervention			Preintervention		Postintervention			Pre	Post
		Mean	Standard Deviation	Mean	Standard Deviation	*p* value	Mean	Standard Deviation	Mean	Standard Deviation	*p* value	*p* value	*p* value
Weight [kg]	P	72.18	±15.21	71.53	±13.95	0.36	73.1	±18.75	74.83	±15.97	0.55	0.91	0.64
BMI [kg/m^2^]	P	26.94	±5.56	26.70	±5.04	0.29	27.8	±6.14	28.51	±5.27	0.53	0.44	0.46
Scapular strength [kg/f]	NP	9.55	4.9: 23.75	9.52	4.59: 16.25	-0.34	10.45	4.90: 23.0	11.27	4.90: 26.25	0.50	0.73	0.67
Right-hand grip strength [kg/f]	P	26.63	±10.13	30.50	±10.24	0.15	25.9	±10.77	28.83	±12.74	0.03*	0.88	0.76
Left-hand grip strength [kg/f]	P	25.95	±9.44	28.73	±10.39	0.08	24.7	±10.54	26.13	±13.95	0.51	0.78	0.65
Lumbar strength [kg/f]	P	37.65	±21.71	51.95	±18.96	0.02*	53.1	±35.3	58.14	±30.42	0.34	0.26	0.60

Central and variation: parametric paired t-tests P: mean and (standard deviation); Nonparametric tests NP: median and |minimal: maximum| Wilcoxon test, *p<0.05 and 95% CI.

**Table 4:** Comparisons of physical and cognitive performance of people with multiple sclerosis.

Variables		Experimental (EG)					Control (CG)					Intergroups	
												(EG) x (CG)	
		Preintervention		Postintervention			Preintervention		Postintervention			Pre	Post
		Mean	Standard Deviation	Mean	Standard Deviation	*p* value	Mean	Standard Deviation	Mean	Standard Deviation	*p* value	*p* value	*p* value
TUG [score]	NP	2.00	1.00: 2.00	1.00	1.00: 2.00	0.046*	1.00	1.00: 2.00	1.00	1.00: 2.00	0.32	0.11	0.61
TUG [s]	P	11.15	±2.46	9.35	±2.02	0.01*	9.89	±1.85	8.70	±1.51	0.04*	0.23	0.44
EDSS [scale]	NP	2.00	1.00: 5.00	2.00	0.00: 5.00	0.22	1.50	150: 5.00	1.50	1.50: 5.00	0.317	0.83	0.83
Locom. speed. [s]	P	519.40	±139.64	393.70	±119.49	0.03*	455.2	±83.54	454.1	±140.58	0.97	0.25	0.33
Dual task 1 [s]	NP	55.50	31.62: 197	43.19	31.9: 139.8	0.028*	40.56	29.92: 95.66	43.91	29.92: 114	0.31	0.12	0.74
Dual task 2 [s]	NP	53.00	37.22: 285	39.32	27.3: 210.9	0.013*	36.84	29.67: 80.40	45.30	34.75: 98	0.07	0.022*	0.33

Central and variation: parametric tests, arithmetic mean and (standard deviation); Nonparametric tests NP: median and |minimal: maximum| *p<0.05 and 95% CI. Time Up and Go (TUG); Expanded Disability Status Scale (EDSS); Locomotion speed (Locom. Speed); s: seconds.

**Table 5:** Comparisons of stabilometric measures on the force platform at the standing time of people with multiple sclerosis.

Variables		Experimental (EG)					Control (CG)					Intergroups	
												(EG) x (CG)	
		Preintervention		Postintervention			Preintervention		Postintervention			Pre	Post
		Mean	Standard Deviation	Mean	Standard Deviation	*p* value	Mean	Standard Deviation	Mean	Standard Deviation	*p* value	*p* value	*p* value
CoP Trajectory covered [cm]	P	38.24	±6.2	26.64	±7.72	0.01*	32.01	±7.82	38.88	±18.26	0.19	0.07	0.07
CoP AP amplitude [cm]	P	11.35	±1.85	8.76	±1.71	0.01*	10.45	±3.06	9.66	±3.15	0.53	0.44	0.44
CoP ML amplitude [cm]	NP	3.38	2.94: 8.32	3.2	1.53: 8.62	0.19	4.96	3.1: 6.78	5.99	|.2: 18.7	0.051	0.10	0.01*
Elliptical area [cm^2^]	NP	35.95	27.6: 108.1	36.7	14.49: 82.73	0.45	68.85	20.98: 96.78	62.16	29.79: 262	0.77	0.14	0.09
CoP AP Median frequency [Hz]	P	1.00	±0.37	1.56	±0.48	0.02*	1.66	±0.44	1.3	±0.31	0.050	0.01*	0.18
CoP ML median frequency [Hz]	P	1.30	±0.51	1.71	±0.4	0.02*	1.47	±0.49	1.59	±0.32	0.62	0.48	0.48

Central and variation: parametric tests, arithmetic mean and (standard deviation); Nonparametric tests NP: median and |minimal: maximum| sig: significance *p<0.05 and 95% CI; Center of Pressure (CoP); Anteroposterior (AP); Medial-Lateral (ML); Hertz (Hz).

**Table 6:** Comparisons of stabilometric measures on the force platform at the sitting time of people with multiple sclerosis.

Variables		Experimental (EG)					Control (CG)					Intergroups	
												(EG) x (CG)	
		Preintervention		Postintervention			Preintervention		Postintervention			Pre	Post
		Mean	Standard Deviation	Mean	Standard Deviation	*p* value	Mean	Standard Deviation	Mean	Standard Deviation	*p* value	*p* value	*p* value
CoP Trajectory covered [cm]	P	45.19	±15.63	29.84	±7.65	0.01*	32.16	±6.06	31.19	±6.12	0.68	0.03*	0.68
CoP AP amplitude [cm]	P	12.40	±2.7	9.94	±2.41	0.02*	11.84	±3.17	9.53	±2.16	0.04*	0.68	0.70
CoP ML amplitude [cm]	P	4.17	±2.8	3.47	±1.37	0.29	5.48	±2.05	5.01	±1.69	0.65	0.21	0.04*
Elliptical area [cm^2^]		56.92	±43.47	42.36	±16.48	0.29	70.13	±46.02	55.48	±19.9	0.32	0.53	0.13
CoP AP median frequency [Hz]	NP	0.8	0.80: 1.40	1.25	0.64: 2.15	0.047*	1.35	0.8: 2.6	1.22	0.58: 1.35	0.66	0.08	0.22
CoP ML median frequency [Hz]	P	1.31	±0.37	1.53	±0.39	0.27	1.88	±0.63	1.62	±0.41	0.30	0.038	0.64

Central and variation: parametric tests, arithmetic mean and (standard deviation); Nonparametric tests NP: median and |minimal: maximum| sig: significance *p<0.05 and 95% CI; Center of Pressure (CoP); Anteroposterior (AP); Medial-Lateral (ML); Hertz (Hz).
